# Long-Term Follow-up and Mortality Rate of Patients of the Randomized Freeway Stent Study

**DOI:** 10.1007/s00270-023-03646-0

**Published:** 2024-01-25

**Authors:** Klaus Hausegger, Wiebke Kurre, Henrik Schröder, Johannes Dambach, Stefanie Stahnke, Christian Loewe, Karl Schürmann, Roman Fischbach, Jochen Textor, Stephan Schäfer, Stephan Müller-Hülsbeck

**Affiliations:** 1 Institut Für Diagnostische und Interventionelle Radiologie, Klinikum Klagenfurt am Wörthersee, Feschnigstraße 11, 9020 Klagenfurt, Austria; 2 Institut Für Diagnostische und Interventionelle Radiologie und Neuroradiologie, Klinikum Passau, Innstraße 76, 94036 Passau, Germany; 3 Gemeinschaftspraxis für Radiologie, Neuroradiologie & Zentrum für Minimal Invasive, Therapie am Jüdischen Krankenhaus Berlin, Heinz-Galinski-Str. 1, 13347 Berlin, Germany; 4 Eurocor Tech GmbH, In den Dauen 6a, 53117 Bonn, Germany; 5 Kardiovaskuläre und Interventionelle Radiologie, Medizinische Universität Wien, Währinger Gürtel 18-20, 1090 Vienna, Austria; 6 Institut für Diagnostische, Interventionelle Radiologie St.-Johannes-Hospital Dortmund, Johannesstraße 9-17, 44137 Dortmund, Germany; 7 Radiologie, Neuroradiologie und Nuklearmedizin, Asklepios Klinik Altona, Paul-Ehrlich-Str. 1, 22763 Hamburg, Germany; 8 Abteilung für Radiologie Gemeinschaftskrankenhaus Bonn, St. Elisabeth/St. Petrus/St. Johannes gGmbH, Bonner Talweg 4-6, 53113 Bonn, Germany; 9 Klinik für Diagnostische und Interventionelle Radiologie, Klinikum St. Elisabeth Straubing GmbH, St.-Elisabeth-Str. 23, 94315 Straubing, Germany; 10 Institut Für Diagnostische und Interventionelle Radiologie und Neuroradiologie, Diakonissenkrankenhaus Flensburg, Knuthstraße 1, 24939 Flensburg, Germany

**Keywords:** Drug-eluting balloon, Long-term benefit, Mortality, Paclitaxel, PAD

## Abstract

**Purpose:**

This follow-up study was designed as a reopen of the completed Freeway Stent Study and collected mortality and clinical outcome data for at least 5 years after enrollment to evaluate long-term patient safety and treatment efficacy. The primary study enrolled 204 patients with stenosis or occlusion in the superficial femoral artery and proximal popliteal artery. Patients were randomized to primary nitinol stenting followed by standard PTA or primary nitinol stenting followed by FREEWAY™ paclitaxel-eluting balloon PTA.

**Methods:**

Previous patients were recontacted by phone or during a routine hospital visit, and medical records were reviewed. Vital and clinical status information was collected.

**Results:**

No increased late mortality was observed at 5 years, with an all-cause mortality rate of 12.0% in the FREEWAY drug-eluting balloon group versus 15.0% in the non-paclitaxel PTA group. No accumulation of any cause of death was observed in either group, nor was there any correlation with the dose of paclitaxel used. Freedom from clinically driven target lesion revascularization at 5 years was significantly higher in the FREEWAY drug eluting balloon group (85.3%) compared to standard PTA group (72.7%) Log-rank *p* = 0.032.

**Conclusion:**

The safety results presented support the recent conclusions that the use of paclitaxel technology does not lead to an increase in mortality. At the same time, the efficacy results clearly demonstrate that the potential benefits of drug-eluting balloon treatment are maintained over a 5-year period.

**Graphical Abstract:**

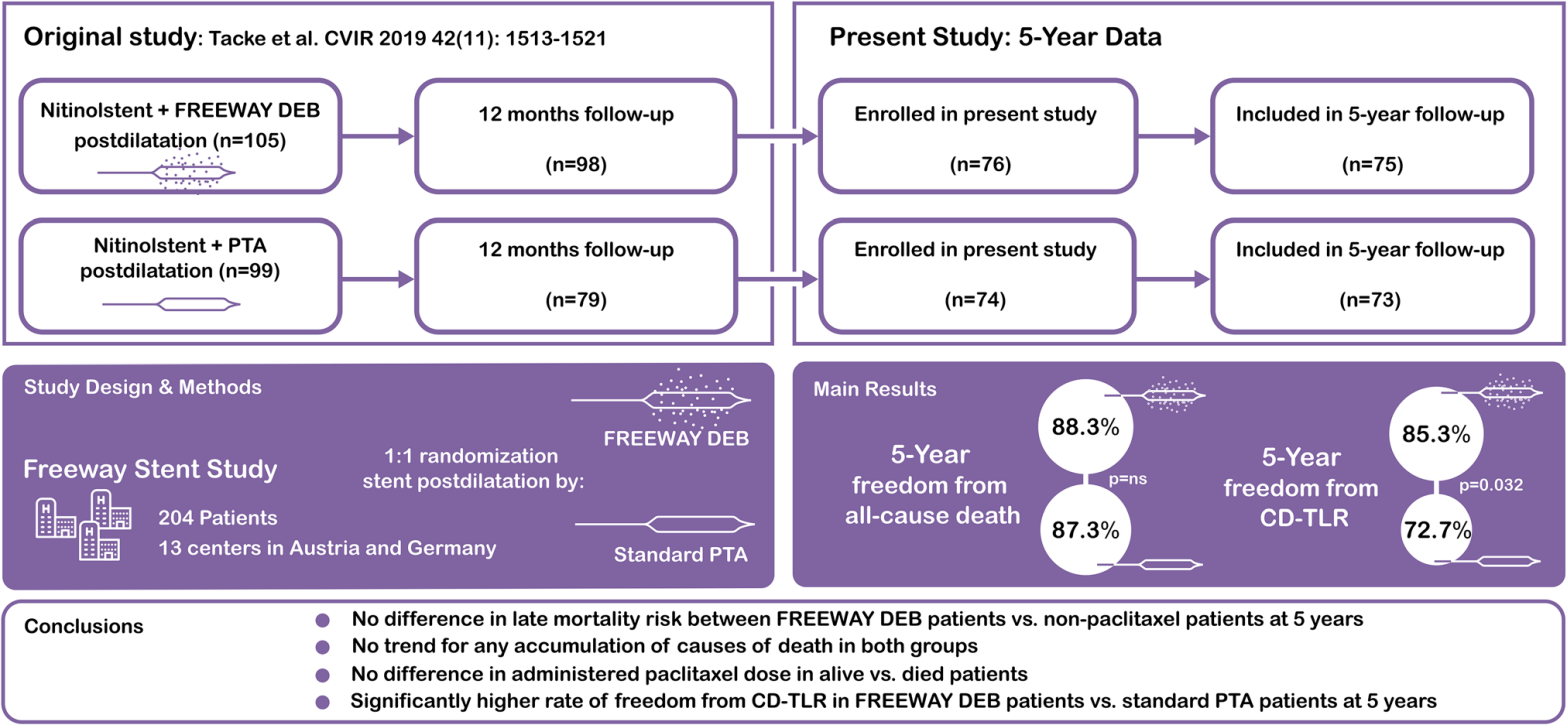

## Introduction

The use of drug-eluting balloons (DEBs) in the treatment of peripheral artery disease (PAD) has been shown to be a safe and effective endovascular treatment option for both de novo and in-stent restenosis lesions. Several randomized trials have demonstrated the superiority of paclitaxel-eluting balloons over standard PTA at least through mid-term follow-up (up to 3 years) [1–6]. However, in 2018, a meta-analysis by Katsanos et al. [7] found a potential signal for an increased late mortality in PAD patients between 2 and 5 years after intervention in association with the use of paclitaxel-eluting balloons and stents. The finding of this association sparked concern and scientific debate about study design, the continued use of paclitaxel devices, and the strengths, limitations, and data quality of studies such as the 2018 meta-analysis. The FDA responded with advisory letters to healthcare providers [8–10] and an expert panel meeting [11]. Further reanalysis of the underlying patient data from the originally included randomized trials by FDA and VIVA physicians [12, 13] replicated the signal, but with a lowered hazard ratio and without the finding of paclitaxel dose dependence. It has been argued that since no plausible biological mechanism could be identified to explain the mortality at the doses of paclitaxel administered, care should be taken not to incorrectly attribute the observed patterns of cause of death to paclitaxel devices [12]. Several subsequent large real-world data analyses [14–16] as well as large new randomized data analyses [17, 18] could not find a mortality signal for paclitaxel-eluting devices at mid-term follow-up of 2–4 years. In this context, it was decided to reopen the completed Freeway Stent Study [19] and to collect long-term mortality and clinical outcome data for at least 5 years after enrollment to contribute to the current discussions.

## Material and Methods

The completed Freeway Stent Study was a prospective, open, randomized trial [19] that enrolled 204 patients at 13 centers in Germany and Austria with stenosis or occlusion in the superficial femoral artery (SFA) and proximal popliteal artery (PI) segment. Patients were randomized to primary nitinol stenting followed by standard plain balloon angioplasty (PTA) or primary nitinol stenting followed by FREEWAY™ paclitaxel-eluting balloon PTA (Eurocor Tech GmbH, Bonn, Germany). Patients were followed at 6 and 12 months. The present study is a reopening of the earlier Freeway Stent Study described above and was initiated to collect long-term data of at least 5 years (60 months). The study protocol was approved by local ethics committees and conducted in accordance with Good Clinical Practice guidelines and the Declaration of Helsinki. Former patients were recontacted and interviewed either by telephone or during a routine check-up in the hospital. Information on patients’ vital status and current clinical status was supplemented by information from hospital medical records. Patient information included date of follow-up and date and cause of death (if patient died), date of potential repeat paclitaxel device interventions, date of potential target lesion revascularization, and occurrence of major adverse events (MAEs). Required MAE data included occurrence of study target lesion stent stenosis or stent thrombosis and occurrence of minor or major amputation.

### Outcome Measures

The primary outcome measure was all-cause mortality at 5 years. Secondary outcome measures were all-cause mortality at 2–4 years and the rate of clinically driven target lesion revascularization at 2–5 years. In addition, stent stenosis or very late stent thrombosis, amputation, assessment of cause of death, and paclitaxel dose calculation with correlation to mortality, all at 5 years, were included.

### Statistics

Mortality was analyzed using the proportional method and by Kaplan Meier estimate. In the proportion method, the total number of patients who died was divided by all patients with available 5-year follow-up data. Kaplan–Meier survival analysis was used to evaluate time-to-event data and group comparisons (log-rank test) for patient survival (freedom from all-cause death), clinically driven target lesion revascularization (freedom from CD-TLR), and stent stenosis or very late stent thrombosis events. For the Kaplan–Meier analyses, all former patients in the primary study who were lost to follow-up in the present study were included as censored at 1 year. Patients who received a paclitaxel device during follow-up but were previously randomized to the non-paclitaxel PTA arm were censored at the time of paclitaxel administration. Unlike the Kaplan–Meier analysis, the proportion method does not allow for patient censoring, so these patients had to be excluded for the proportional mortality analysis. Summary statistics were expressed as hazard ratios (HR) and associated 95% control intervals (CI) or risk ratios and associated 95% CI’s for specific follow-up times. Continuous data are presented as mean ± standard deviation; hypotheses were tested with unpaired t test. Categorical data are presented as absolute patient number and percentage; hypotheses were tested using Fisher’s exact test with two-tailed *P* value calculation. The statistical significance was determined as *p* ≤ 0.05.

### Drug Dose

The dose of paclitaxel administered during the primary study was calculated based on the surface area and concentration (3.0 µg/mm^2^) of the FREEWAY™ drug-eluting balloons used. The dose of further paclitaxel treatments (balloons and stent) during the follow-up period was also calculated based on the devices and paclitaxel concentrations used.

## Results

### Patient Characteristics

**Fig. 1:**
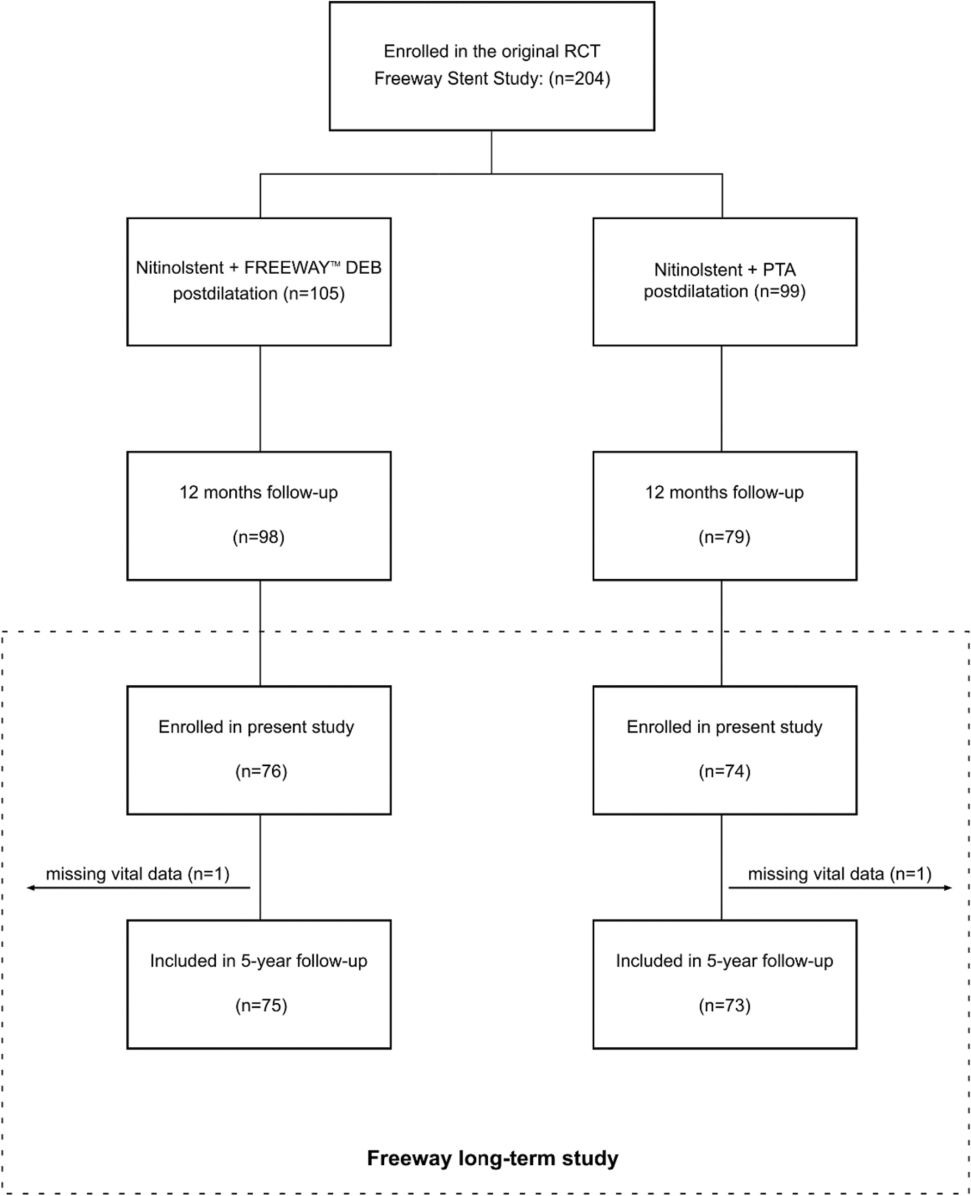
The study flowchart shows that 204 patients were enrolled in the primary Freeway Stent study between 2010 and 2016 and received nitinol stent implantation and FREEWAY postdilation or nitinol stent implantation and uncoated PTA balloon postdilation. The primary study had a follow-up at 12 months. For the current study, 148 patients (75 and 73) were included for analysis of 5-year data

In this study, 151 patients (76 from the FREEWAY group and 74 from the PTA group) were enrolled between December 2021 and March 2023 (Fig. 1). One patient in the FREEWAY and one in the PTA group were excluded due to missing information in vital status at 5 years. A total of 13 patients in the non-paclitaxel PTA group received a paclitaxel device during follow-up and were treated as censored at the time of drug administration in the Kaplan–Meier analysis. These 13 patients were excluded from the non-paclitaxel group in the proportionate mortality analysis. At the time of study query, all patients had reached or exceeded the target minimum follow-up of 5 years. Demographic characteristics collected in the primary study and selected for patients who were enrolled in this long-term follow-up showed no significant differences between the two study groups (Table 1).

**Table 1:** Demographic characteristics collected in the primary study are shown for patients enrolled in this long-term follow-up and show no significant differences between both study groups

	FREEWAY DEB (*N* = 75)	PTA (*N* = 73)	*p* value (ns > 0.05)
Male	76.0%	78.1%	ns
Age	65.5 ± 9.5 years	64.8 ± 9.3 years	ns
Diabetes	26.7%	27.4%	ns
History of PAD	32.0%	49.3%	ns
History of CAD	26.7%	20.5%	ns
Smoking	89.3%	84.9%	ns
Hyperlipidemia	60.0%	58.9%	ns
Hypertension	74.7%	76.7%	ns

*DEB *  drug eluting balloon,* PTA* percutaneous transluminal angioplasty,* PAD*  peripheral artery disease,* CAD* coronary artery disease

### The Primary Outcome

**Fig. 2:**
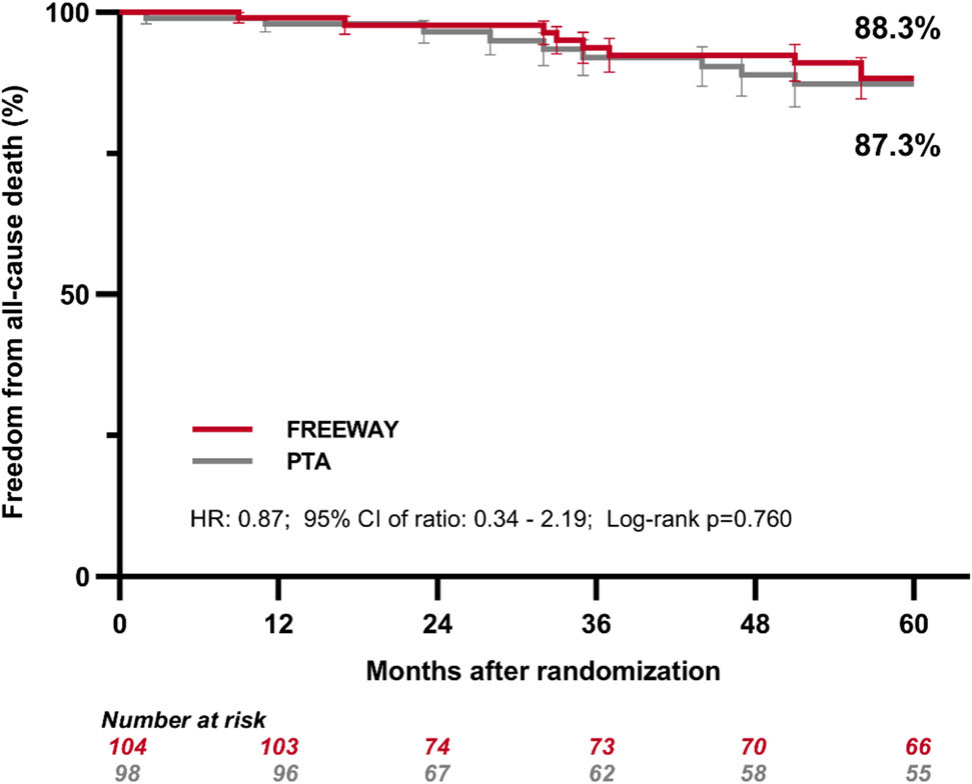
Freedom from all-cause death / patient survival by Kaplan Meier estimate at 5 years after inclusion

There was no significant difference in all-cause mortality at 5 years (FREEWAY DEB group 12.0 vs. 15.0% non-paclitaxel PTA group; risk ratio (RR), 0.81; 95% CI 0.35–1.90 calculated by proportion method). Accordingly, Kaplan–Meier analysis showed that 5-year freedom from all-cause death was 88.3% in the FREEWAY DEB group versus 87.3% in the non-paclitaxel PTA group HR: 0.87; 95% CI 0.34–2.19, log-rank *p* = 0.760 (see Fig. 2, Table 2).

**Table 2:** Safety data at 5 years after inclusion by Kaplan Meier estimate

Freedom from:	FREEWAY DEB (%)	PTA (%)	*Δ* KM rate (%)	log-rank *p* Value
All-cause death	88.3	87.3	1.0	0.760
Stent stenosis or very late stent thrombosis	82.0	77.1	4.9	0.260
Minor or major amputation	97.1	100.0	2.9	0.217

*DEB* drug eluting balloon,* PTA * percutaneous transluminal angioplasty,* Δ KM rate* difference in Kaplan Meier estimates between treatment and control group

### Secondary Outcomes

**Fig. 3:**
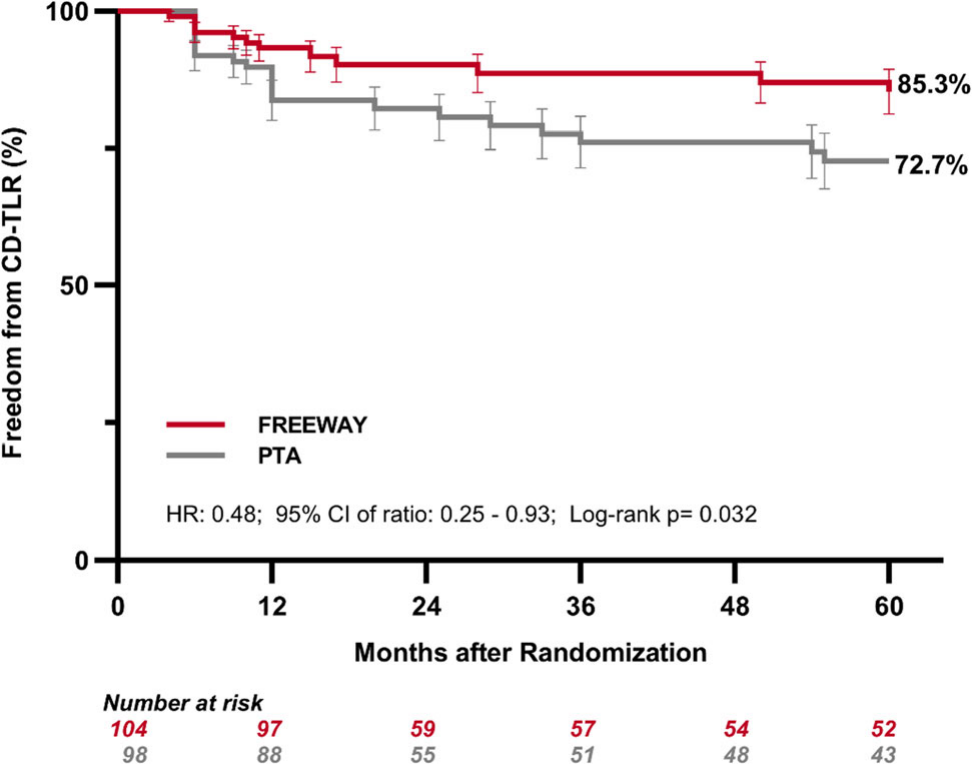
Freedom from clinically driven target lesion revascularization (CD-TLR) by Kaplan Meier estimate at 5 years after inclusion

Mortality rates at 2, 3, and 4 years showed no difference between DEB and standard PTA group: 2.7 vs. 5.0% at 2 years (RR 0.53; 95% CI 0.09–3.08), 6.7 vs. 10.0% at 3 years (RR 0.67; 95% CI 0.21–2.07), and 8.0 vs. 15.0% at 4 years (RR 0.53; 95% CI 0.20–1.40) (proportion method). Freedom from clinically driven target lesion revascularization (TLR) was significantly higher at 5 years after randomization in patients treated with FREEWAY DEB compared to those treated with standard PTA: 85.3 vs. 72.7%; HR: 0.48; 95% CI 0.25–0.93, log-rank *p* = 0.032 (Fig. 3). Freedom from CD-TLR from 2 to 4 years was 90.2 vs. 82.2%; log-rank *p* = 0.078 at 2 years and 88.6 vs. 76.1%; log-rank *p* = 0.026 at 3 and 4 years. Rate of combined stent stenosis and very late stent thrombosis at 5 years was similar in both groups with 18.0% (FREEWAY) and 22.9% (PTA) (Table 2). At 5 years, one major and one minor amputation were reported in the DEB group and none in the PTA group (Table 2).

### Paclitaxel Dose

The mean paclitaxel dose administered per patient showed no significant differences between living and deceased patients up to 5 years (8388 µg ± 7227 µg [*N* = 66] vs. 10,847 µg ± 7140 µg [*N* = 9]; *p* = 0.340) and longer up to the time of study data collection between 2021 and 2023 (8737 µg ± 7655 µg [*N* = 57] vs. 8513 µg ± 5834 µg [N = 18]; *p* = 0.908).

### Causes of Death

Causes of death reported at the time of the survey were categorized as cardiovascular, respiratory, infectious, cancer, other, or unknown if no information was available. Multiple causes were possible per patient. The reported data showed no trend toward an accumulation of causes of death in either group (Table 3).

**Table 3:** Causes of death

Cause of death	FREEWAY DEB (*N* = 18)	PTA (*N* = 17)
Cardiovascular	2	2
Respiratory	0	2
Infection	1	0
Cancer	4	8
Other	0	2
Unknown	11	7

Table shows causes of death for all cases reported until the study query

*DEB* drug eluting balloon,* PTA*  percutaneous transluminal angioplasty

## Discussion

In this 5-year long-term follow-up of the previously completed Freeway Stent Study, no increased mortality was observed in patients treated with FREEWAY paclitaxel-eluting balloons compared to patients who did not receive paclitaxel. Mortality at 2–5 years did not differ significantly between paclitaxel-eluting balloon PTA and standard PTA patients (Fig. 2). Several subsequent large real-world data analyses [14–16] as well as large new randomized data analyses [17, 18] have not shown a mortality signal for paclitaxel-eluting devices at mid-term follow-up of 2–4 years being in line with the results of the present long-term study. Furthermore, even when two different methods of mortality analysis are used, Kaplan–Meier estimation and proportional analysis, the results differ only slightly in the mortality rates found, and both have a hazard ratio or risk ratio of less than 1.

To date, there are few randomized trials of drug-eluting balloons in the femoropopliteal arteries with long-term follow-up of up to 5 years. Randomized 5-year data were presented in the IN.PACT SFA trial [20] and the Thunder trial [21], both of which were included in the 2018 meta-analysis [7], as well as the Levant 2 trial [22], which was not included with 5-year data at that time, the AcoArt 1 trial [23] and the EffPac trial [24]. Additional large, randomized data for femoropopliteal arteries up to 4 years were presented in the ILLUMENATE Pivotal Study [25], the SWEDEPAD interim analysis [17] and the Voyager PAD trial [18].

The IN.PACT SFA trial [20] had a 2:1 randomization and included 331 patients; it showed a significantly higher mortality rate for the DEB group at 2 and 3 years, but no significant difference in rates at 4 and 5 years. Using the “proportion method” for better comparability, as performed in the reanalysis by Holden et al. [26], the Levant 2 trial (2:1 randomization, 532 patients) also showed no significant difference in 5-year mortality between paclitaxel and non-paclitaxel group. Holden et al. argue that the theory of a causal relationship between dose and mortality is refuted when mortality and paclitaxel dose from the IN.PACT and Levant2 DEB trials are compared with those from the Zilver PTX trial, finding that the highest dose was associated with the lowest mortality and the lowest dose was associated with the highest mortality [26]. Paclitaxel dose analysis in the present study was performed with patient-level paclitaxel data similar to the analysis in Donas et al. [27] and found no correlation between paclitaxel dose and mortality in patients who died versus those who were still alive. Similar to the results presented here, the 5-year data from the AcoArt 1 trial (200 patients) and the EffPac trial (171 patients) showed no significant difference in mortality rates between the DEB and the uncoated balloon group [23, 24]. The ILLUMENATE pivotal trial (371 patients) showed a nearly identical 4-year mortality rate between the paclitaxel and control arm [25] and the intermittent claudication group (> 800 patients) in the SWEDEPAD interim analysis at 4 years or the VOYAGER PAD results (1342 patients) at 3 years [17, 18] found no significant difference in mortality rates for patients treated with paclitaxel devices. In the present study no particular clustering of reported causes of death was observed for the paclitaxel or non-paclitaxel treatment groups. After reviewing several of the new large real-world data and randomized trials mentioned above and others, the FDA issued an update letter to health care providers on July 11, 2023, [28] concluding that the totality of the data now available does not support an excess risk of mortality with paclitaxel-coated devices. In the same way, the authors of a CIRSE expert opinion paper [29] conclude that "a robust body of evidence now exists to refute the existence of a long-term mortality signal associated with PCDs" and further support that the favorable results seen with the use of these devices in terms of primary patency and TLR rate should ensure the routine use of these devices in the femoropopliteal area. Thus, the long-term mortality data presented here for the FREEWAY™ drug-eluting balloon join a number of other recently published randomized or real-world mid- and long-term data studies and expert conclusions that show no difference in mortality rates between paclitaxel and non-paclitaxel PTA balloon treatment.

The efficacy outcome in this study shows that 5-year freedom from CD-TLR was significantly higher in patients treated with FREEWAY DEB compared to standard PTA balloon treatment (85.3 vs. 72.7% log-rank *p* = 0.032). The delta of CD-TLR between the two groups was 12.6% at 5 years. The 5-year delta for CD-TLR found in the EffPac trial [24] was slightly lower compared to the present results with *Δ* = 8.4% (82.1 vs. 73.7% *p* = 0.050), the delta found in the IN.PACT study [20] was similar at *Δ* = 10.1%, higher in the Thunder trial (*Δ* = 34.8%) [21] or in the AcoArt 1 trial (*Δ* = 18.4%) [23] and lower at *Δ* = 5.9% in the ILLUMENATE trial at 4 years [25]. However, comparisons between trials should be interpreted with caution because not only differences in patient population or procedure (e.g., stenting, bail-out stenting, or no stenting) but also differences in data analysis (e.g., proportional analysis or ITT analysis without exclusion of patients lost to FU) may affect the absolute number and the delta between groups.

## Limitations

The original study was not powered for statistical analysis of 5-year mortality or TLR between the two treatment arms. The majority of patients were contacted by phone only. A clinical visit was not mandatory in this study design.

## Conclusion

In conclusion, new randomized trial data and large real world data analyses as well as the results of the present study did not find a mortality signal as seen in the 2018 meta-analysis data. To date, no plausible biological mechanism has been identified to explain the mortality, and no cause of death was found to be associated with the use of paclitaxel at doses administered with drug-eluting devices. Furthermore, the efficacy results clearly demonstrate the clinical benefit of drug-eluting balloon treatment over a 5-year period.
